# Beyond Publication Counts: Trends in Neurosurgical Publishing via a Retrospective Analysis of the Arms Race Control Score

**DOI:** 10.1227/neuprac.0000000000000262

**Published:** 2026-07-02

**Authors:** Clayton R. Baker, Sameer Sundrani, Astoria Chao, Austin Hilvert, Lohit K. Velagapudi, Lola B. Chambless

**Affiliations:** 1 Vanderbilt University School of Medicine, Nashville, Tennessee, USA;; 2 Department of Neurological Surgery, Vanderbilt University Medical Center, Nashville, Tennessee, USA

**Keywords:** Neurosurgical education, Bibliometrics, Publication arms race

## Abstract

**BACKGROUND AND OBJECTIVES:**

Research productivity has become increasingly emphasized in neurosurgery residency applications, and applicant publication counts continue to rise. The Arms Race Control Score (ARCS) was proposed to standardize research effort, but its temporal stability and predictive value for future output remain unclear. Among board-certified US neurosurgeons, we sought to retrospectively examine how the ARCS has evolved temporally across 20 residency cohorts (1995-2014) and evaluate the predictive value of the ARCS on postresidency research productivity.

**METHODS:**

Publication data for neurosurgeons certified by the American Board of Neurological Surgery between 2009 and 2024 were extracted from Scopus. Leveraging large-language model analysis of Scopus's document type, title, journal, and abstract, publications were categorized based on ARCS criteria. For each surgeon, ARCS was calculated at the time of residency start. Distributions were analyzed across 5-year residency cohorts. Correlations and multivariable regressions tested associations between ARCS and long-term outputs (postresidency publication total, citation total, and h-index), adjusting for years since residency graduation and pre-residency publication count.

**RESULTS:**

This cohort included 1776 board-certified neurosurgeons who began residency between 1995 and 2014 and had a total of 96 249 publications. ARCS steadily increased over time (*P* < .001), with the most recent cohort (2010-2014) having a significantly higher ARCS than all previous cohorts on post hoc comparisons. ARCS was positively correlated with all long-term research metrics, even when controlling for duration since residency graduation (all *P* < .001). In multivariable regression, both ARCS and pre-residency publication count predicted postresidency publication total and h-index. However, only ARCS predicted postresidency citation total.

**CONCLUSION:**

In a retrospective cohort, ARCS moderately increased in more recent residency cohorts and is a modest, independent predictor of long-term research productivity. These findings support ARCS as a complementary metric—alongside holistic review—for interpreting applicant research beyond raw counts.

**ABBREVIATIONS:**
ARCS: arms race control score
LLM: large language model
PES: publication effort score.

Neurosurgery is among the most competitive medical specialties in the United States, with 476 applicants applying for 268 spots in 2025.^1^ In considering potential applicants, programs consistently report research as an important factor.^2,3^ This emphasis has amplified since the US Medical Licensing Exam Step 1 became pass-fail, removing an objective screening metric.^4,5^ This growing focus on research output has created a publication “arms race,” as applicants across medical specialties increasingly produce more research.^6-9^

Within neurosurgery, bibliometric literature has explored research productivity trends. Increased research output is not limited to applicants: this trend has also been observed in residents and attendings.^10-16^ The number of coauthors on publications has also been increasing, a trend observed in tandem with increasing publication counts.^15,17^ As coauthorship rises, individual contribution becomes harder to discern, especially for nonlead authors. Although the total number of publications on a residency application has traditionally been an evaluative tool, given these trends, it may not be informative in evaluating individual contributions to a project.^18,19^ To address these concerns, the “Arms Race Control Score” (ARCS) was recently proposed by Bowers et al^18^ as one such metric to standardize research productivity. By scaling publications broadly by overall “effort” (based on study type) and weighing based on authorship position, ARCS creates one potential objective measure of research contribution.

While current work rightly focuses on exploring the implications of this score on current applicants, there may be additional utility drawn from retrospectively applying this score to previous applicants for the purposes of external validation of the predictive value of ARCS.^18^ Indeed, research performed before residency may not just reflect current interest in a field but also serve as an important predictor of future scholarly contribution. Previous bibliometric work has explored the early signals, such as training environment and early career research volume, that may relate to later productivity, both through residency and into later career practice.^14,15^ However, these studies have relied on raw publication counts, which do not account for the type of scholarly work or the individual's role in producing it. A standardized, effort-weighted system, which scales publications by study type and authorship position, offers a framework to test whether the quality of early research, not just quantity, predicts long-term output. In the context of continuously rising publication counts, it is important to evaluate a standardized metric, such as ARCS, for both stability over time and predictive value for future research output. Thus, among a cohort of board-certified US neurosurgeons, we sought to retrospectively (1) examine how ARCS has evolved temporally across 20 residency cohorts (1995-2014) and (2) evaluate whether ARCS predicts postresidency research productivity.

## METHODS

### Data Collection

From the American Board of Neurological Surgery's “Find a Neurosurgeon” website,^20^ all US-based neurosurgeons and board certification date were extracted. Using the Scopus API (Elsevier), neurosurgeons were automatically name-matched by their first and last name, including middle initial when available.^21,22^ When multiple profiles appeared, the top-returned profile was selected. For each matched profile, h-index was recorded. Subsequently, document-level metadata for all Scopus-indexed items linked to that profile were extracted, which included: Title, Authors, Journal, Abstract, Publication Date, Document-Type, and Total Citations. Additional information regarding each neurosurgeon's training was collected, including the start and end year of residency from publicly accessible sources including institutional websites, Doximity profiles (Doximity, Inc.), US News Health listings (US News & World Report), and LinkedIn profiles (LinkedIn Corporation) in Spring 2025. If no definitive classification was possible (e.g., irreconcilable training dates), the neurosurgeon was excluded from analyses. Impact factor for journals was extracted from Clarivate's Journal Citation Reports (Clarivate Plc).^23^ Board-certified neurosurgeons were identified with general Scopus profiles in Fall 2024, and additional Scopus document level information and Journal Citation Reports were accessed in Spring 2025. This study used publicly available professional-profile data and bibliometric metadata; therefore, institutional review board approval and informed consent were not required.

### Inclusion Criteria

Neurosurgeons who were board certified in 2009 or later and started residency between 1995 and 2014 were included. Neurosurgeons without a Scopus profile at the time of data collection were excluded. To mitigate erroneous matches, all neurosurgeons with outlier ARCS values, denoted as those outside the IQR fence (Quartile 1 − 1.5*IQR to Quartile 3 + 1.5*IQR) were manually inspected for bibliometric accuracy and excluded if Scopus profile linkage was inaccurate.

### Independent Variables

For each neurosurgeon, ARCS before residency start was calculated according to the proposed formula from Bowers et al,^18^ with a general overview available in **Supplemental Digital Content 1** (**Supplemental Table 1**, http://links.lww.com/NS9/A117). First, the publication effort score (PES) was determined for each Scopus document by leveraging the Scopus document type. For items labeled as an article, article in-press, or review, a large language model (LLM, OpenAI's “gpt-3.5-turbo”^24^) was prompt engineered to leverage the item's title, abstract, and journal to provide a classification: case report, case series (<30 vs ≥30 patients), clinical study, basic science study, cadaveric study, narrative review, systematic review, or meta-analysis. A random sample of 100 LLM classified documents were manually evaluated (C.R.B., A.C.) and specific prompts are detailed in **Supplemental Digital Content 1** (**Supplemental Table 1**, http://links.lww.com/NS9/A117). For high effort publications (PES = 4), PES score was modified by +1 if the journal's 5-year impact factor was >10.2 based on a threshold from the original formulation. For each neurosurgeon, each of their indexed documents' PES was normalized by their authorship position (i.e., PES/authorship position). Following the initial ARCS calculation, which does not include “Minimal Effort Publications,” all normalized scores of at least 1 were summed to calculate a given neurosurgeon's ARCS. Only indexed documents published before the neurosurgeon's residency start year were considered in the score.

### Outcome Variables

Primary outcome was total citations, h-index, and postresidency publications. To account for exposure time, corrected per-year values were calculated by dividing by the years from residency end to 2025 [i.e., Corrected Post-Residency Publications = Post-Residency Publications/(2025 – Residency End Year)].

### Statistical Analysis

Descriptive statistics were reported as mean ± SD or median (IQR) for continuous variables and count (%) for categorical variables. ARCS was grouped in 5-year bins across residency-start cohorts (1995-1999, 2000-2004, 2005-2009, 2010-2014). Comparative analysis was performed between these 5-year cohorts with Kruskal-Wallis test, followed by Dunn post hoc testing with Holm correction if the former demonstrated significance. Correlational analysis with Spearman ρ and univariate regression were performed to assess ARCS relationship to outcome variables. This was repeated with pre-residency publication count to benchmark with another common metric. Multivariable regression used ARCS, pre-residency publication total, and duration from residency end to 2025 as covariates. A *P* value of <.05 was considered significant. All analyses were performed using Python 3.12 (Python Software Foundation).

## RESULTS

Of 2514 publicly listed neurosurgeons who were board-certified in 2009 or later, there were 2146 with Scopus profiles. Of these, 1970 had verifiable training dates (92.2%), and 1933 neurosurgeons were confirmed to begin residency between 1995 and 2014. After excluding 158 for incorrect matching, there were a total of 1776 neurosurgeons with 96 249 Scopus-indexed publications. On average, this cohort was board certified 11 years from the start of residency and 5 years from the end of residency. Neurosurgeons had an average of 2.6 ± 6.3 publications before residency, 11.7 ± 18.7 publications during residency, and 39.9 ± 75.3 publications after residency. The average ARCS for this cohort was 3.9 ± 9.2. Complete overview of the cohort is presented in Table 1. LLM classification of documents was accurate (86/100 (86%) agreement with human classifier), and of the 14 disagreements, only 3 would have affected PES or ARCS. The remainder would not have affected PES and were predominantly misclassification between clinical and basic science or narrative and systematic reviews. There was also near-perfect agreement between human raters (Cohen kappa = 0.97).

**TABLE 1.** Overview of Neurosurgeon Cohort

Neurosurgeons, n	1776
Residency start year, med (IQR)	2006 (2002-2010)
Residency end year, med (IQR)	2013 (2008-2017)
Board year, med (IQR)	2018 (2014-2021)
Time from residency start to board year (y), med (IQR)	11.0 (10.0-12.0)
Time from residency end to board year (y), med (IQR)	5.0 (4.0-6.0)
Pre-residency scopus-indexed items, mean ± SD	2.6 ± 6.3
During-residency scopus-indexed items, mean ± SD	11.7 ± 18.7
Postresidency Scopus-indexed items, mean ± SD	39.9 ± 75.3
ARCS, mean ± SD	3.9 ± 9.2

ARCS, arms race control score; med, median.

### Temporal Analysis

**FIGURE 1.**
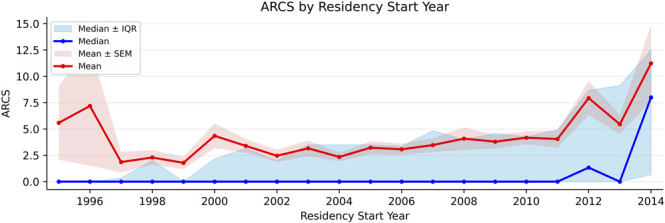
Mean ARCS relative to residency start year. ARCS, arms race control score.

Across 5-year residency start cohorts, ARCS increased significantly over time (*P* < .001, Table 2, Figure 1). While the median ARCS was 0 across all cohorts, both the mean and upper bound of the IQR rose steadily, with the latter increasing from 0 (1995-1999) in the earliest cohort to 7.3 in the latest cohort (2010-2014). On post hoc testing, the earliest cohort (1995-1999) had significantly lower ARCS than all subsequent cohorts, while the most recent cohort (2010-2014) had significantly higher ARCS than all prior cohorts (all *P* ≤ .025, **Supplemental Digital Content 2**, **Supplemental Table 2**, http://links.lww.com/NS9/A118).

**TABLE 2.** 5-Year Residency Start Year Comparison of Average ARCS

	1995-1999	2000-2004	2005-2009	2010-2014	*P*-value
ARCS, mean ± SD; median (IQR)	2.7 ± 10.1; 0.0 (0.0-0.0)	3.1 ± 7.5; 0.0 (0.0-3.3)	3.5 ± 7.4; 0.0 (0.0-4.0)	5.6 ± 11.8; 0.0 (0.0-7.3)	<.001

ARCS, arms race control score.

### Long-Term Productivity Metrics

**FIGURE 2.**
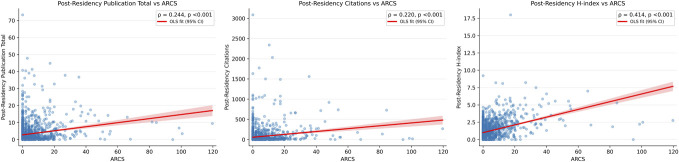
Scatterplots of ARCS and long-term productivity metrics corrected for duration from residency end year to 2025. ARCS, arms race control score.

There was a significant positive relationship with ARCS and all long-term productivity metrics: postresidency publications (ρ = 0.202, *P* < .001), postresidency citations (ρ = 0.174, *P* < .001), and h-index (ρ = 0.377, *P* < .001). Even when correcting for duration since residency graduation to present, the correlation between ARCS to postresidency publications (ρ = 0.244, *P* < .001), postresidency citations (ρ = 0.220, *P* < .001), and h-index (ρ = 0.414, *P* < .001) remained significantly positive (Figure 2). A similar positive relationship was also observed with pre-residency publication total. When correcting for duration since residency graduation to present, the significant positive relationships remained with postresidency publications (ρ = 0.238, *P* < .001), postresidency citations (ρ = 0.212, *P* < .001), and h-index (ρ = 0.425, *P* < .001). More information regarding these correlations is included in **Supplemental Digital Content 3** (**Supplemental Table 3**, http://links.lww.com/NS9/A119).

Univariate analysis found that both ARCS and pre-residency publication total were independently and positively associated with each long-term productivity metric (Table 3). In multivariate regression, both ARCS (B: 0.834, 95% CI: 0.200-1.467, *P* = .010) and pre-residency publication total (B: 1.041, 95% CI: 0.116-1.967, *P* = .027) were independent, positive predictors of postresidency publication total. While ARCS (B: 56.183, 95% CI: 33.154-79.211, *P* < .001) also independently predicted postresidency citation total, pre-residency publication total (B: −7.072, 95% CI: −40.720 to 26.576, *P* = .680) was no longer a significant predictor. Both ARCS (B: 0.260, 95% CI: 0.154-0.366, *P* < .001) and pre-residency publication total (B: 0.500, 95% CI: 0.346-0.655, *P* < .001) were independent, positive predictors for h-index. Regression statistics are presented in Table 3.

**TABLE 3.** Regression Analysis of ARCS and Pre-Residency Publications to Long-Term Productivity Metrics

	Univariate		Multivariate	
	B (95% CI)	*P*-value	B (95% CI)	*P*-value
Postresidency publication total				
ARCS	1.251 (0.876-1.627)	<.001	0.834 (0.200-1.467)	.010
Pre-residency publication total	1.729 (1.181-2.276)	<.001	1.041 (0.116-1.967)	.027
Postresidency citation total				
ARCS	45.170 (31.419-58.921)	<.001	56.183 (33.154-79.211)	<.001
Pre-residency publication total	46.650 (26.500-66.799)	<.001	−7.072 (−40.720 to 26.576)	.680
H-index				
ARCS	0.514 (0.451-0.577)	<.001	0.260 (0.154-0.366)	<.001
Pre-residency publication total	0.761 (0.669-0.852)	<.001	0.500 (0.346-0.655)	<.001

ARCS, arms race control score.

## DISCUSSION

Amid rising publication numbers in neurosurgery residency applicants, the ARCS was proposed to help standardize evaluation of individual research effort.^18^ In our retrospective analysis, we observed a gradual rise in ARCS among incoming neurosurgery residents over time and externally validate that ARCS broadly correlates with postresidency research productivity.

Across 20 residency cohorts, ARCS was lowest in the late 1990s and increased over subsequent cohorts, with the most pronounced rise in the early 2010s. The median ARCS remained 0 across all cohorts—indicating that most neurosurgeons in this era had no pre-residency publications meeting the ARCS effort threshold. However, the observed temporal increase was driven by a steady rise in proportion of neurosurgeons with substantive early-career research, reflected by the rising mean and upper IQR over time. These findings are consistent with the broader trends in research output among neurosurgery applicants, which in part motivated the development of ARCS.^6,7,18^

In this national cohort of board-certified neurosurgeons, ARCS was consistently, though modestly, associated with postresidency productivity. A high pre-residency ARCS corresponded to increased postresidency publications, postresidency citations, and h-index. When these values were corrected to control for neurosurgeons who finished residency earlier and had more time to publish research, ARCS remained positively associated with these long-term research metrics. Furthermore, this correction only modestly improved correlation values, suggesting that time-in-practice only accounts for some variability in the relationship between ARCS and long-term research output. Previous work has demonstrated that training environment, academic culture, and early career research contributions may help predict long-term research contribution.^14,15^ This study corroborates such previous literature and has found that pre-residency publication volume may also modestly correspond to long-term productivity. In multivariable regressions controlling for time from residency end to 2025, both ARCS and pre-residency publication volume were independent predictors for postresidency publication volume and h-index, whereas only ARCS independently predicted postresidency citation amount. Though these metrics are all interconnected and size-dependent (i.e., generally increase with more publications), it is possible that citations may grow exponentially more from a few high impact publications.^25^ In this sense, pre-residency publication volume may less accurately identify postresidency citation amount, when compared with a metric such as ARCS, which more closely focuses on impact and effort.

For residency selection committees, these findings suggest that ARCS may offer incremental value beyond raw publication counts when reviewing applications. Effort-weighted work, even early in medical training, may reflect research behaviors—such as initiating, sustaining, and completing projects—that are foundational to long-term productivity. Used alongside holistic review of personal statements, letters of recommendation, and other experiences, ARCS may help program directors distinguish applicants whose early scholarly involvement is more likely to translate into sustained research contribution after residency. Even so, ARCS is just one metric to evaluate applicants and is focused on scientific contribution. Importantly, it fails to recognize differences in opportunity among applicants. Previous work has established that those at more resourced medical schools may more easily contribute to research, in part due to more institutional resources for mentorship and funding.^26-28^ Moreover, other contributions, such as those to open-source technology or innovation, are not readily accounted for in ARCS. Although basic science often takes more time to complete, ARCS provides equal weight to basic and clinical research.^29^ By not accounting for time investment, ARCS may undervalue those with deep basic science training or provide unfair comparison for those with substantial research experience but relatively fewer publications. While scientific contribution is an important aspect of neurosurgery, many surgeons also contribute through their clinical and surgical impact as well as other leadership endeavors. Concerns over ARCS becoming just another score to “game” may also support a more qualitative evaluation of an applicant's research portfolio.^18,30^ Although such approaches are harder to standardize or automate, future work could explore the relationship of objective metrics to subjective evaluations.

### Limitations

There are several limitations associated with this study. Importantly, the residency cohorts studied here (1995-2014) largely predate the most recent acceleration in applicant publishing. In the past 10 years, and particularly in the pass/fail era of Step 1, publication counts among residency applicants have risen.^7,28,31^ While the end of our analysis cohort captures the beginning of this observed trend, it cannot definitively apply to current cohorts. Although ARCS was demonstrated to predict actual research interest in previous years, publishing behaviors may have evolved in the last decade (i.e., box-checking). Extrapolating these findings to future applicant cohorts may not be accurate, and future work should evaluate more recent graduating cohorts as they enter practice and accrue postresidency outcomes.

Residency start and end years were collected from publicly available sources, not standardized databases. Publication metrics were obtained from Scopus and author matching relied on automated, top-result name-based linkage to enable large-scale retrieval.^15^ This mimics prior large-scale bibliometric strategies in neurosurgery^15^ but can introduce author disambiguation errors through both false-positive matches (linking to the wrong author) and false-negative exclusions (failing to match a profile). To mitigate false-positives, outlier neurosurgeons were manually reviewed and 158 (8.2%) of initially matched profiles were excluded for incorrect Scopus linkage. To assess false-negative bias, a random subset of 50 neurosurgeons who were excluded due to absent automated Scopus matches were searched via Google Scholar and Google; 86% (43/50) had at least one identifiable publication, suggesting the final cohort may underestimate overall publication activity. While incorporating middle initials increased matching stringency, it may have excluded some neurosurgeons whose Scopus profiles did not include middle initials or those with middle name changes. Prior bibliometric work suggests Scopus may be more accurate for individualized analysis,^16^ although future work should consider multiple databases (Google Scholar, PubMed, etc.) and more intensive matching strategies leveraging institutional affiliation, publication history, and coauthor networks. In addition, LLMs were used to classify certain Scopus-indexed document types (e.g., article, in-press, review), and some classification error is possible despite prompt tailoring and strong performance on manual evaluation. Finally, demographic variables including gender or race/ethnicity were not available from publicly reported databases and were not included in analysis. These factors, along with medical school and residency characteristics such as funding, dedicated research time, and academic rank, are known to influence research opportunity and productivity.^12-14^ Individuals with name changes may be undercaptured in bibliometric databases, which could affect representativeness.^32,33^ Future work should integrate demographic and program-level data into bibliometric and effort-weighted publication analyses.

## CONCLUSION

In this cohort of American Board of Neurological Surgery–certified neurosurgeons, higher ARCS modestly predicted postresidency publications, citations, and h-index. These findings support ARCS as a useful complement within holistic review of neurosurgery residency applicants.

## Supplementary Material

## Supplemental Digital Content

**Supplemental Digital Content 1: Supplemental Table 1.** Publication effort score (PES) matching based on Scopus document type and Arms Race Control Score (ARCS) calculation process; * and **: Separate prompting for classification based on item's title, abstract, and journal; ^: Items with PES value of 4 were eligible for +1 if published in journal with 5-year impact factor greater than 10.2. **Supplemental Digital Content 2: Supplemental Table 2.** Post hoc Dunn test with Holm correction between 5-year residency start year cohorts and arms race control score (ARCS). **Supplemental Digital Content 3: Supplemental Table 3.** Spearman analysis for arms race control score (ARCS) and pre-residency publication total and long-term productivity metrics, including correction for duration from residency end to 2025.
