# Supplementary material for: Beyond Publication Counts: Trends in Neurosurgical Publishing via a Retrospective Analysis of the Arms Race Control Score
Source: Neurosurg Pract. 2026 Jul 2;7(4):e000262. doi: 10.1227/neuprac.0000000000000262 (PMC13322472; doi:10.1227/neuprac.0000000000000262)
Supplement: Supplementary file 1 [file neuopen-7-e000262-s001.docx]

*Supplemental Table 1:* Publication Effort Score (PES) matching based on Scopus document type, Large language model (LLM) prompts, and Arms Race Control Score (ARCS) calculation process; ^*^ and ^**^: Separate prompting for classification based on item’s title, abstract, and journal; ^^^: Items with PES value of 4 were eligible for +1 if published in journal with 5-year impact factor greater than 10.2

| Scopus Document Type | Automated Classification | | PES Value |
| --- | --- | --- | --- |
| Erratum | *NA* | | 0 |
| Note |  |  | 0 |
| Book (non-peer reviewed) |  |  | 0 |
| Chapter (non-peer reviewed) |  |  | 0 |
| Report |  |  | 0 |
| Short Survey |  |  | 0 |
| Conference Review |  |  | 0 |
| Conference Paper |  |  | 0 |
| Retracted Document |  |  | 0 |
| Editorial |  |  | 1 |
| Letter |  |  | 1 |
| Data Paper |  |  | 4^^^ |
| Article  Article In-Press | *LLM Classification^*^* | |  |
|  | Case Report | | 2 |
|  | Case Series (<30 patients) | | 3 |
|  | Case Series (≥30 patients) | | 4^^^ |
|  | Clinical Science | | 4^^^ |
|  | Basic Science | | 4^^^ |
|  | Cadaveric | | 4^^^ |
| Review | *LLM Classification^**^* | |  |
|  | Narrative Review | | 3 |
|  | Systematic Review | | 3 |
|  | Meta-Analysis | | 4^^^ |
| LLM Classification*^*^* | | | |
| For Articles or Articles in Press | | System Role: “You are a research assistant trained to classify scientific papers based on their metadata.”  Prompt: “Title: {title}; Journal: {journal}; Abstract: {abstract}; Is this a (A) Case report, (B) Case series < 30 patients, (C) Case series >= 30 patients, (D) Clinical Science Research Study, (E) Basic Science Research Study, (F) Cadaveric study? Just reply with one letter (A–F), and no explanation. Example Output: A” | |
| For Reviews | | System role: “You are a research assistant trained to classify scientific papers based on their metadata.”  Prompt: “Title: {title}; Journal: {journal}; Abstract: {abstract }; Is this a (A) narrative review, (B) systematic review, or (C) meta-analysis? If it is both a systematic review and meta-analysis, then classify it as a meta-analysis (C). Just reply with one letter (A-C), and no explanation. Example Output: A” | |
| ARCS Calculation | | | |
| Publication Value Unit (PVU) = PES/(Authorship Position) | | ARCS = ∑ PVU ⎢PVU ≥ 1 | |
