# Supplementary material for: Beyond Publication Counts: Trends in Neurosurgical Publishing via a Retrospective Analysis of the Arms Race Control Score
Source: Neurosurg Pract. 2026 Jul 2;7(4):e000262. doi: 10.1227/neuprac.0000000000000262 (PMC13322472; doi:10.1227/neuprac.0000000000000262)
Supplement: Supplementary file 2 [file neuopen-7-e000262-s002.docx]

*Supplemental Table 2:* Post-hoc Dunn’s Test with Holm correction between 5-year Residency Start Year Cohorts and Arms Race Control Score (ARCS)

| Cohort 1 | Cohort 1 Mean | Cohort 1 Med (IQR) | Cohort 2 | Cohort 2 Mean | Cohort 2 Med (IQR) | p-value |
| --- | --- | --- | --- | --- | --- | --- |
| 1995–1999 | 2.7 | 0.0 (0.0 - 0.0) | 2000–2004 | 3.1 | 0.0 (0.0 - 3.3) | 0.025 |
| 1995–1999 | 2.7 | 0.0 (0.0 - 0.0) | 2005–2009 | 3.5 | 0.0 (0.0 - 4.0) | 0.001 |
| 1995–1999 | 2.7 | 0.0 (0.0 - 0.0) | 2010–2014 | 5.6 | 0.0 (0.0 - 7.3) | <0.001 |
| 2000–2004 | 3.1 | 0.0 (0.0 - 3.3) | 2005–2009 | 3.5 | 0.0 (0.0 - 4.0) | 0.089 |
| 2000–2004 | 3.1 | 0.0 (0.0 - 3.3) | 2010–2014 | 5.6 | 0.0 (0.0 - 7.3) | <0.001 |
| 2005–2009 | 3.5 | 0.0 (0.0 - 4.0) | 2010–2014 | 5.6 | 0.0 (0.0 - 7.3) | 0.001 |
