# Supplementary material for: Beyond Publication Counts: Trends in Neurosurgical Publishing via a Retrospective Analysis of the Arms Race Control Score
Source: Neurosurg Pract. 2026 Jul 2;7(4):e000262. doi: 10.1227/neuprac.0000000000000262 (PMC13322472; doi:10.1227/neuprac.0000000000000262)
Supplement: Supplementary file 3 [file neuopen-7-e000262-s003.docx]

*Supplemental Table 3:* Spearman Analysis for Arms Race Control Score (ARCS) and Pre-Residency Publication Total and Long-term Productivity Metrics, including correction for duration from Residency End to 2025

|  | Post-Residency Publications | | Corrected Post-Residency Publications | |
| --- | --- | --- | --- | --- |
|  | ρ | p-value | ρ | p-value |
| ARCS | 0.202 | <0.001 | 0.244 | <0.001 |
| Pre-residency Publication Total | 0.193 | <0.001 | 0.238 | <0.001 |
|  | Post-Residency Citations | | Corrected Post-Residency Citations | |
|  | ρ | p-value | ρ | p-value |
| ARCS | 0.174 | <0.001 | 0.220 | <0.001 |
| Pre-residency Publication Total | 0.164 | <0.001 | 0.212 | <0.001 |
|  | H-Index | | Corrected H-Index | |
|  | ρ | p-value | ρ | p-value |
| ARCS | 0.377 | <0.001 | 0.414 | <0.001 |
| Pre-residency Publication Total | 0.383 | <0.001 | 0.425 | <0.001 |
